# Supplementary material for: Learning curve and surgical outcome of robotic assisted colorectal surgery with ERAS program
Source: Sci Rep. 2022 Nov 29;12:20566. doi: 10.1038/s41598-022-24665-w (PMC9709162; doi:10.1038/s41598-022-24665-w)
Supplement: Supplementary file 1 — Supplementary Information 1. [file 41598_2022_24665_MOESM1_ESM.zip › LSC Raw data-Ñ[▒K/141/N_ñw╝╨░O▒Kñσ.pdf]

W83 / W83

臺中榮民總醫院

[redacted]

## 基本資料

- 室別： 二醫手術室-31室，手術科別： CRS，手術類別： 常規，日期： 2021/10/13
- 進入時間： 0935 ； 麻醉開始時間： 0940 ； Time Out時間： 1045 ； 劃刀時間： 1050 ； 手術結束時間： 1445 送出時間： 1505 ；
- 麻醉方式： GE
  診斷：rectum ca
  手術名稱：1.Low anterior resection with colo-anal anastomosis2.Ileostomy3.Laparoscopy4.
  手術局部用藥：nil
  術前預防性抗生素：
  - 抗生素名稱一： Veterin 1GM IVD/由麻醉護理人員給予
  - 時間： 1030
  - 記錄者： 陳昱穎

  ，追加預防性抗生素：
- 手術醫師： 林俊余
- 麻醉醫師： VS 沈靜慧
- 
  - 刷手護士A ： 吳明柔，時間： 0935
  - 流動護士A ： 彭馨慧，時間： 0935，接替流動一： 陳昱穎，時間： 1500

---

## 皮膚狀況

A-V Shunt： 無
術前： 完整
記錄者： 上次簽章: 彭馨慧
術後： 完整
記錄者： 陳昱穎

---

## 手術準備

手術臥式 單一手術臥式

- 
  - 手術臥式： 截石臥式
  - 使用附件： 溫毯 38 ℃，頭圈，腳架
  - 皮膚準備： 10%Aq Betadine，Hibiscrub，2% Chlorhexidine
  - 電燒： 使用電燒: 電極片位置： 其它： 使用床上迴路電極墊，記錄者： 上次簽章: 彭馨慧

止血帶T： 無

---

## 植入物

- 植入物名稱/號碼/數量/部位：Ethicon ligaclip/20釘/pelvic/LOT V94J2K
  Ethicon endo GIA EC45A/伍釘/colon
  Ethicon CDH29A/壹/colon

，記錄者： 上次簽章: 彭馨慧

---

## 標本送檢

- 細菌培養 無
- 細胞學 無
- 冰凍切片 無
- 病理標本
  組織名稱一：Rectum
  ，
  組織名稱二：Distal cut end
  ，
  組織名稱三：IMA root
  ，更多組織名稱
  - 

  - 組織名稱四： IMV root
- 截肢 無

標本交付 標本： ? 袋 , 標本： 零 盒 , 標本： 壹 瓶 , 細胞學： 零 管 , Culture Tube： 零 管 , 被交付醫師： R 林志安 ,

- 記錄者： 上次簽章: 彭馨慧
- 時間： 1455

---

## 傷口術後敷料

傷口敷料： 傷口敷料： 無

- 請於圖上標示部位：

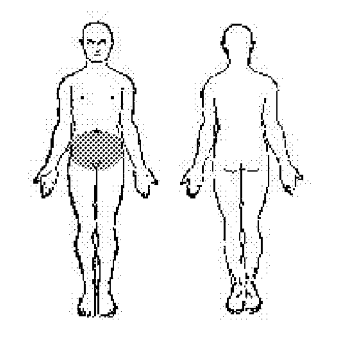

腹部 叁切口

- 傷口敷料： menpor覆蓋

---

## 導管

| 導管 | 天數 | 導管資訊 | 插入日期 | 插入時間 | 更換日期 | 拔除日期 | 拔除時間 | 拔除原因 | 預計拔除/更換 | 預計拔除/更換日期 |
|---|---|---|---|---|---|---|---|---|---|---|
| Sump NG Tube(1) | 1 | 號碼:16 Fr.; 部位:胃; | 2021/10/13 | 1015 | | | | | | |
| Foley(1) | 1 | 號碼:16 Fr.; 部位:膀胱; | 2021/10/13 | 1020 | | | | | | |
| Rod,Stoma Supporting (1) | 1 | 部位:小腸; | 2021/10/13 | 1410 | | | | | | |
| A-line(1) | 1 | 號碼:20號 ; 部位:右手; | 2021/10/13 | 1000 | | | | | | |
| Hemo Vac (1) | 1 | 號碼:15 Fr.; 部位:骨盆腔; | 2021/10/13 | 1400 | | | | | | |

---

## 用物計數

- 手術中出血量： 少量 c.c.
- 手術中尿量： 200 c.c.
- 
  - 其它： 無

用物計數： 多切口同時縫合

- 1.器械： 正確 , 2.縫針： 正確 , 3.刀片： 正確 , 4.紗布： 正確 , 5.腹部墊： 未使用 , 6.紗球： 未使用 , 7.PEANUTS ： 未使用 , 8.紗條： 未使用 , 9.絲帶： 正確 , 10.針頭： 未使用 , 11.Vessel loop： 未使用 , 12.Boots： 未使用 , 13.Jelco： 未使用 , 14.棉條： 未使用 ,
  - 第一次：
    - 刷手護士 ： 吳明柔
    - 流動護士： 上次簽章: 彭馨慧
    - 時間： 1350

  ,
  - 第二次：
    - 刷手護士 ： 吳明柔
    - 流動護士： 上次簽章: 彭馨慧
    - 時間： 1400

  ,
  - 第三次：
    - 刷手護士 ： 吳明柔

- 流動護士： 上次簽章: 彭馨慧
- 時間： 1410

## 備註

備註： NIL

- 記錄者： 上次簽章: 彭馨慧
- 時間： 1400

填寫護理人員 陳昱穎

正式摘要

W83 / W83

doctorRank:B
